# Stunting in the first year of life: Pathway analysis of a birth cohort

**DOI:** 10.1371/journal.pgph.0002908

**Published:** 2024-02-16

**Authors:** Martha Mwangome, Moses Ngari, Daniella Brals, Paluku Bawhere, Patrick Kabore, Marie McGrath, James A. Berkley

**Affiliations:** 1 KEMRI/Wellcome Trust Research Programme, Kilifi, Kenya; 2 The Childhood Acute Illness and Nutrition Network, Nairobi, Kenya; 3 Amsterdam Centre for Global Child Health, Emma Children’s Hospital, Amsterdam University Medical Centres, Amsterdam, Netherlands; 4 School of Public Health, Center of Research in Epidemiology Biostatistics and Clinical Research, Université Libre de Bruxelles, Brussels, Belgium; 5 Africa Regional office, World Health Organisation, Brazzaville, Republic of Congo; 6 Emergency Nutrition Network (ENN), Kidlington, Oxfordshire, United Kingdom; 7 Centre for Clinical Vaccinology & Tropical Medicine, University of Oxford, Oxford, United Kingdom; Translational Health Science and Technology Institute, INDIA

## Abstract

Malnutrition among infants aged below 6 months has been largely overlooked creating gaps in our understanding of factors underlying stunting in early infancy. Recent evidence suggests that pre-natal and early childhood factors may contribute more to driving childhood stunting than previously appreciated. The study was set up to examine pathways including parental and household characteristics, birth size and gestation, and illness in infancy with stunting at birth and months 3, 6 and 12 using an a priori hypothesized framework. It was a secondary analysis of a birth cohort of 1017 infants recruited from four health facilities in Burkina Faso and followed up for one year. Structural equation models (SEM) were generated to explore pathways to stunting at birth and months 3, 6 and 12. The prevalence of being stunted at birth and months 3, 6 and 12 was 7.4%, 23%, 20% and 18% respectively. The fractions of month 12 stunting attributable to being stunted at birth, months 3 and 6 were 11% (95%CI 5.0‒16%), 32% (95%CI 22‒41%) and 40% (95%CI 31‒49%) respectively. In the structural equation model, male sex and maternal characteristics had direct effects on stunting at birth and at 3 months, but not subsequently. Premature birth, twin birth and being stunted at a previous time point were directly associated with stunting at months 3, 6 and 12. Both maternal and paternal characteristics were directly associated with preterm birth. Non-exclusive breastfeeding had borderline positive direct effect on stunting at month 6 but not at month 12. The direct and indirect pathways identified in this study highlight the complex interlinks between child, maternal, paternal and household characteristics. Interventions tackling preterm birth, in utero growth, exclusive breastfeeding and maternal wellbeing may reduce stunting in the first year of life.

## Introduction

Globally, 149 million children are stunted (height/length-for-age Z score (LAZ) < -2) and the rate of decline has been unacceptably slow [1]. The international community regards stunting as a global child health and nutrition priority. This is reflected in the United Nations Sustainable Development Goal (SDG) 2.2 to end hunger and all forms of malnutrition by 2030, with a specific target to reduce levels of stunting by 50% [2]. This goal aligns with the target set by the World Health Assembly in 2012 of 40% reduction of stunting by 2025. The Scaling Up Nutrition Movement together with other global initiatives aim to support countries to achieve these targets [2].

Studies exploring prevalence and factors underlying the process of stunting among children under 5 years have predominantly focused on children aged 6–59 months, often overlooking infants under 6 months [3]. This may be due to several factors: nutrition surveys typically only include children 6–59 months of age and primarily gather data on wasting prevalence to inform caseload projections for treatment, practical challenges in measuring length in young infants, and an assumption that infants below the age of 6 months are exclusively breastfed and thus at low risk of nutritional deficit. However, multi-setting breastfeeding data show that whilst overall 44% infants under 6 months are reported to be exclusively breastfed [4], less than 10% exclusive breastfeed continuously from 0 until 6 months of age [5].

Importantly, a significant number of infants are born stunted [3], especially those born preterm (when using WHO growth standards) which are not corrected for gestation [6, 7], suggesting *in utero*, genetic or epigenetic effects as key drivers of early childhood stunting [6]. The proportion of children who are stunted generally increases from birth to 24 months of age. For example, the Malnutrition and Enteric Disease study (MAL-ED) longitudinal birth cohort reported an increase in the prevalence of stunting from 43% shortly after birth to 74% at 24 months [6, 8]. However, the proportion of infants under 6 months who experience stunting in childhood that can be attributed to being stunted at birth is largely unreported.

The WHO 2013 conceptual framework for childhood stunting characterises factors into three main domains: context which includes community and societal factors; causes including household and family, feeding and infection related factors; and short and long term consequences of stunted growth such as health, developmental and economic outcomes [9]. Predominantly, studies that have applied this kind of framework have found child, parental and household factors predict stunting in early childhood [10]. However, as the framework does not describe potential pathways and relationships between factors to ultimately contribute to stunting, limiting application to prioritising and designing interventions.

Recent evidence has raised questions regarding the relative contribution of post-natal infant health and nutrition to early childhood stunting. For example, results from the SHINE trial [11] in Zimbabwe and WASH Benefits trials in Bangladesh and Kenya and testing feeding water and sanitation and hygiene (WASH) interventions found that feeding interventions led to a very small increase in LAZ, while WASH interventions alone were unlikely to reduce stunting [11, 12]. Similar results were reported from an observational study in Gambia [13]. Collectively, these studies have prompted enhancing our understanding of the key drivers of early childhood stunting.

We set out to explore the relationship between birth anthropometry, infant, parental and household characteristics and stunting in the first 12 months of life in a birth cohort in Burkina Faso using a pathway analysis.

## Methods

### Study design & population

The study enrolled pregnant women attending antenatal care (ANC) and their infants at four rural health facilities: Foube, Basma, Barsalogho and Dablo in Barsalogho District, Burkina Faso. Data were collected in 2004, as previously detailed [14, 15]. In brief, the primary birth cohort recruited pregnant women attending ANC regardless of whether they delivered in the health facility or at home and infants were followed-up monthly for one year after birth [16].

### Data sources/measurements

For this study, deidentified data were accessed in 2021 from the primary source. Mother height in cm was measured using a standard wooden stadiometer at the point of recruitment. Maternal age was recorded at time of recruitment (first trimester). Infections in pregnancy were determined either using results of prenatal care examination or outpatient care consultation by the pregnant woman. Access to healthcare was crudely assessed by estimating the distance from residential area of the participant to the health facility. Gestational age was assessed by date of mother’s last menstrual period. In addition, morphological criteria of foetal maturation were used to estimate the term of pregnancy (gestational age) described by Eregie et. al. [17–19]. Data on anthropometric status at birth was collected in the first 2 hours and 48 hours of birth for infants born in hospital and at home respectively. Weight was measured using electronic weighing scales (Seca, Birmingham, UK) to the nearest 10g; Mid-upper arm circumference (MUAC) using non-elastic graded measuring tape (TALC, St Albans, UK) in cm to the nearest 0.1cm; and length using an infantometer measuring board (UNICEF standard design) in cm to the nearest 0.1cm. Anthropometric equipment used in the study were those normally used in health facilities. They were quarterly calibrated and maintained by ministry of health using a standard country protocol. All field workers involved in data collection were qualified health professionals (nurses and midwives) working in public health service. They were trained on standard procedures for anthropometric measurement and pre-tests were carried out in all health centers before the study began. The principal investigator supervised all data collection and carried out monthly quality control on the validity of the anthropometric measurements.

Follow-up infant anthropometry was collected monthly until month twelve at the recruiting health facility. Infants who missed a monthly clinic visit would miss anthropometry for that month but would be actively followed-up in the community to resume clinical visit the next month. During the monthly follow-up visits, history of episodes of illness (cough, fever, diarrhoea) were collected at every visit whilst exclusive breastfeeding and introduction of complementary feeding were collected at the first six monthly visits only. If severe wasting was detected, the infant was referred to the district hospital for treatment according to the WHO protocol in place at that time.

### Quantitative variables

Prematurity was defined as estimated gestational age <37 weeks. Birth and monthly infant LAZ, weight-for-length (WLZ) and weight-for-age (WAZ) z-scores were calculated using WHO 2006 Child Growth Standards (WCGS) for term infants and INTERGROWTH 21^st^ Newborn Growth Standards (INBGS) for preterm infants [20]. Being stunted and underweight were defined as <-2 z-scores for LAZ and WAZ respectively. Being wasted was defined as WLZ <-2 but excluded babies born with length <45cm because the WHO 2006 WLZ standards do not include these values.

### Latent exposure variables

We developed *a priori* conceptual framework (**S1 Fig)** based on previous conceptual path models [9, 21], and examined factors in the following categories: maternal characteristics, paternal characteristics, household characteristics, access to health care, pregnancy characteristics and infant-level factors at follow-up as latent variables. The list of individual variables used to create the latent variables is provided on **S1 Table**. The latent variables were created using confirmatory factor analysis (**S2 Table**) using individual variables shown on **S1 Table**. The outcomes assessed were being stunted at birth, months 3, 6 and 12. The estimated latent variables were included as continuous values. The conceptual framework shows the pathways explored in the structural equation models (SEM).

### Study size

The primary birth cohort comprised 1103 infants, and for this analysis, all 86 deaths were excluded as they occurred prior to the infant reaching 12 months of age when the outcome measurement were assessed leaving 1017 infants for analysis at birth. At months 3, 6 and 12 only children with LAZ data were included in the SEM regression. With a fixed sample of 940 children assessed for stunting at month 12, the study had a post-hoc power >90% and two-tailed alpha of 5% to detect adjusted odds ratio >2.5 of association between born premature and stunting at month 12. Similarly, the sample size was adequate to detect adjusted odds ratio of ≥2.5 of association between born twin and being stunted at month 6 and 12 with a post-hoc power >80% and two-tailed alpha of 5%.

### Statistical methods

Proportions stunted were reported with exact binomial 95% confidence intervals. Continuous variables were either reported as mean (standard deviation) or median (Interquartile range) depending on if they had a normal distribution or were skewed respectively. To estimate the proportion of being stunted at month 12 attributable to being stunted at birth and at months 3 and 6, we used the Greenland and Drescher (1993) method [22].

Confirmatory factor analysis (CFA) was used to create standalone latent variables. The predicted latent variables were fitted using Bernoulli logit model (the binary measured variables), ordinal logit models (ordinal measured variables) and linear models (for continuous measured variables). We used SEM to examine the pathways of association between the measured and latent variables with being stunted at birth and months 3, 6 and 12. We constructed the SEM with stunting status at birth and months 3, 6 and 12 as the main outcomes within generalized SEMs using logistic regression models because being stunted was a binary variable with effects reported as adjusted odds ratios (aOR) with 95% confidence intervals (95%CI). In brief, SEM assumes the model is correctly specified adequate sample size, data are missing at random and multivariate normality. Months 3 and 6 were picked to capture the peak of infancy stunting. All continuous latent variables and measured exposures were included in the final multivariable regression model.

To account for unobserved heterogeneity across the health facilities like the level of care and the correlation in being stunted at birth, months 3, 6 and 12, we conducted a multilevel generalized SEM including the recruitment health centre and the different observation times (birth, month 3, 6 and 12) as a random intercept. We used the Benjamini-Hochberg correction to adjust the P values for multiple comparisons to control the false discovery rate in the SEM [23, 24]. Commonly used SEM goodness of fit indices for continuous data typically use maximum likelihood (ML) normal-theory, however, for categorical outcome models, indices estimated through unweighted least squares or diagonally weighted least squares are not universally accepted and unavailable in STATA software [25], thus, no goodness of fit indices were reported. Statistical analyses were conducted using STATA Version 17.0 (College Station, TX, USA).

### Ethical considerations

The primary birth cohort and subsequent data analysis was approved by Burkina Faso Ministry of Health in 2003 (approval number: 1014) according to national procedure. Written informed consent to participate was provided by the infants’ mothers. If severe wasting was detected, the infant was referred to the district hospital for treatment according to the WHO protocol in place at that time. For this analysis data were accessed in June 2017 and since then it has been used to publish several papers [14, 15].

### Results

#### Participants

**Fig 1:**
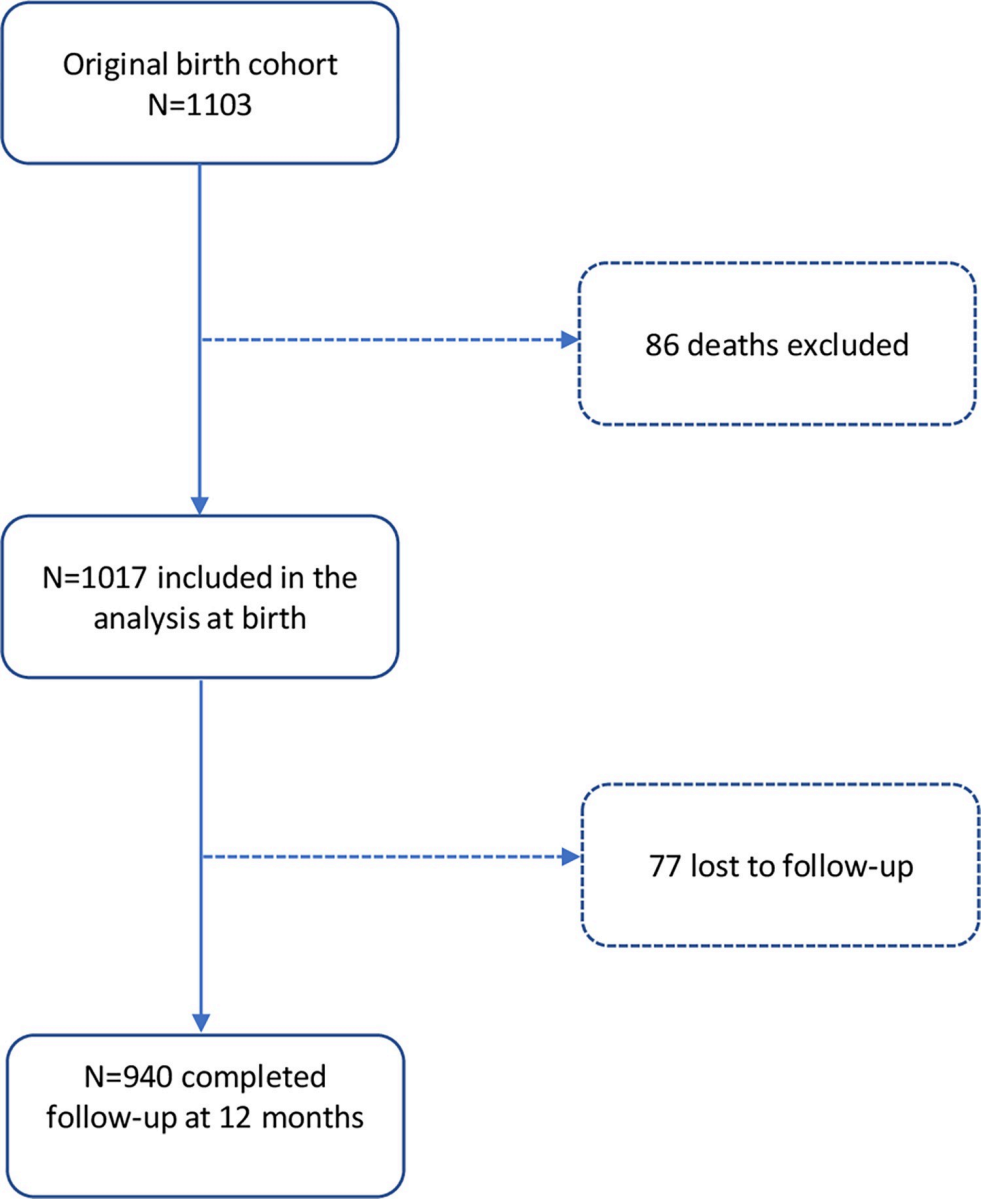
Study participants flowchart.

Amongst the 1017 infants included in the study after excluding perinatal deaths (**Fig 1**), 522 (51%) were male. The median gestational age was 38.9 (IQR 38.2 to 40.2) weeks. Forty-eight (4.7%) infants were born premature and 189 (19%) were low birth weight (LBW). Of the 48 infants born premature, 34 (71%) were also LBW. A total of 454 (45%) infants were born in a health facility, while 399 (39%) and 164 (16%) were born at home with and without a community-based assistant present respectively. The birth characteristics of the total 1103 infants enrolled in the birth cohort and the 1017 infants included in this analysis were similar (**Table 1**).

**Table 1:** Infants and maternal characteristics at time of birth and during follow-up.

	All infants (N = 1,103)	Infants assessed in this study (N = 1,017)
**Demographics**		
Sex		
Male	570 (51)	522 (51)
Gestation age in weeks; median [IQR]	38.9 (38.2–40.2)	38.9 (38.2–40.2)
Born premature*	62 (5.6)	48 (4.7)
Born twins	39 (3.5)	28 (2.8)
Born low birth weight^#^	227 (21)	189 (19)
Place of birth		
Health facility	492 (45)	454 (45)
Home with CBA	432 (39)	399 (39)
Home with no CBA	179 (16)	164 (16)
Recruitment health centre		
Basma	416 (38)	387 (38)
CMA	320 (29)	301 (30)
Dablo	286 (26)	252 (25)
Foube	81 (7.3)	77 (7.6)
**Birth anthropometry (**mean ±sd)		
Length (cm), mean ±sd	48.9 ± 2.6	49.0 ± 2.4
Weight (kg); mean ±sd	2.8 ± 0.5	2.8 ± 0.4
MUAC in cm; mean ±sd	10.2 ± 1.1	10.3 ± 1.0
LAZ; mean ±sd	-0.22 ± 1.3	-0.18 ± 1.2
WLZ; mean ±sd	-1.35 ± 1.6	-1.35 ± 1.6
WAZ; mean ±sd	-0.95 ± 1.0	-0.94 ± 0.9
Stunted; N (%, 95%CI)	89 (8.1, 6.5 to 9.8)	75 (7.4, 5.8 to 9.2)
Underweight; N (%, 95%CI)	149 (14, 12 to 16)	122 (12, 10 to 14)
Wasted; N (%, 95%CI)	327 (31, 29 to 34)	303 (31, 28 to 34)
**Child follow-up characteristics**		
Exclusive breastfeeding		
≥3 months	773 (70)	742 (73)
Up to 3 months	278 (25)	246 (24)
None from birth	52 (4.7)	29 (2.9)
Follow-up illness (No. of children with at least one episode)		
Fever	777 (70)	741 (73)
Diarrhoea	507 (46)	483 (47)
Cough	544 (49)	521 (51)
**Maternal characteristics**		
Mother age; median (IQR) years	25 (20−30)	25 (20−30)
Illiterate	854 (77)	779 (77)
Number of ANC visits		
None	60 (5.4)	52 (5.1)
1 to 3	1024 (93)	947 (93)
≥ 4	19 (1.7)	18 (1.8)
Mother height at delivery; median (IQR) cm	163 (159 to 168)	163 (159 to 168)
**Paternal characteristics**		
Father age; median (IQR) years	39 (31 to 49)	39 (31 to 49)
Father illiterate	568 (52)	514 (51)
Father Body Mass Index (BMI)		
<18.5	108 (9.8)	98 (9.6)
18.5 to 25	902 (82)	829 (82)
≥25.0	93 (8.4)	90 (8.6)
**Household characteristics**		
Wealth quantiles		
Quintile 1 (most assets)	222 (20)	207 (20)
Quintile 2	222 (20)	207 (20)
Quintile 3	218 (20)	201 (20)
Quintile 4	227 (21)	213 (21)
Quintile 5 (Least assets)	214 (19)	189 (19)
Distance to nearest health facility (km)		
< 5Km	406 (39)	374 (39)
5Km	633 (61)	585 (61)

*gestational age <37 weeks, #birth weight <2.5kg, CBA: community based assistant, MUAC: mid-upper arm circumference, LAZ: length-for-age z-score, WLZ: weight-for-length z-score, WAZ: weight-for-age z-score, sd: standard deviation, results are N (%) unless specified, asserts used to calculate wealth quantiles are: bicycle, motorcycle, car, cart, plough, donkey, goat, sheep, pig, cow, radio, TV, type of the house (mud or bricks).

Mothers were a median age of 25 (IQR 20 to 30) years old at recruitment, with median height 163 (IQR 159 to 168) cm. Only 18 (1.8%) mothers attended at least four ANC visits. Seven hundred and seventy-nine (77%) mothers were illiterate, and 374 (39%) households were <5 km from the nearest health facility.

A total of 988 (97%) infants had exclusive breastfeeding reported at birth, 614 (60%) at month 3 and 404 (40%) at month 6. The number of infants in follow-up at each month up to month twelve is shown on **S3 Table.** A total of 77 (7.6%) were not assessed at month 12 but their baseline characteristics were not significantly different from the 940 children who completed 12 months follow-up **(Fig 1 and S4 Table).**

During follow up during the first year of life, 741 (73%) infants had a reported at least one episode of fever, while 521 (51%) and 483 (47%) had reported illnesses with cough and diarrhoea respectively (**Table 1 and S2 Fig**).

### Prevalence of stunting

The mean birth length, weight and MUAC were 49.0 (SD 2.4) cm, 2.80 (SD 0.4) kg and 10.3 (SD 1.0) cm respectively (**Table 1**). Forty-five (4.4%) infants had birth length <45cm. The mean birth LAZ was -0.18 (SD 1.2). The mean WAZ and WLZ were -0.94 (SD 0.9) and -1.35 (SD 1.6) respectively.

The number and proportions stunted, underweight and wasted at birth were 75 (7.4% (95%CI 5.8 to 9.2%)), 122 (12% (95%CI 10 to 14%)) and 303 ((31% (95%CI 28 t 34%)) respectively. Among the 75 infants classified as stunted at birth, 44 (59%) were born LBW and 3 (4.0%) were born premature.

**Fig 2:**
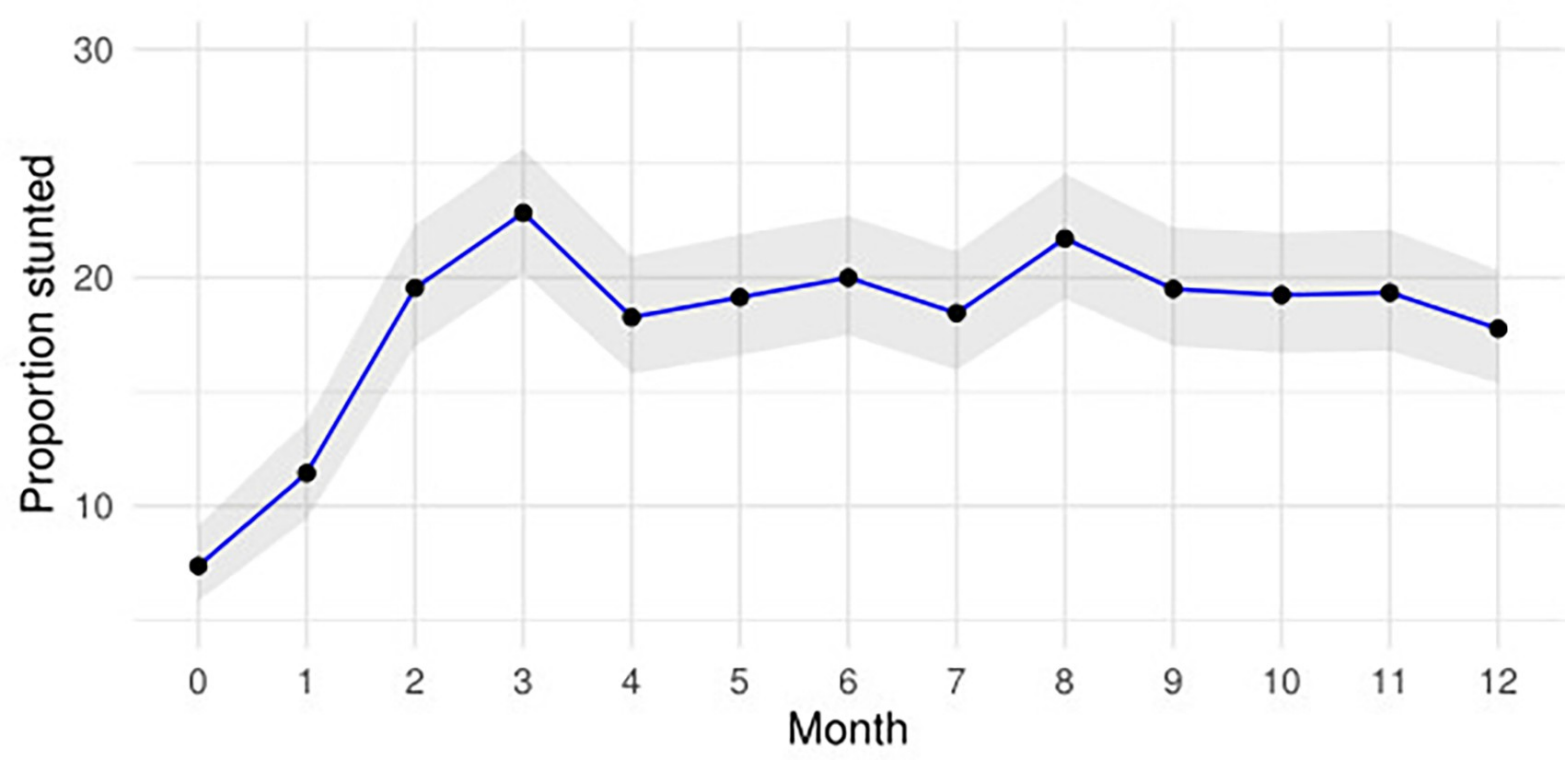
Monthly proportion of children stunted from birth to month twelve of age. The shaded region shows the 95% confidence intervals of the proportion.

Mean LAZ declined from -0.18 (SD 1.2) at birth to -0.72 (SD 1.8) at month 3, -0.77 (SD 1.6) at month 6 and -1.18 (SD 1.2) at month 12. The number (proportion) of infants stunted at months 3, 6 and 12 were 214 (23%), 187 (20%) and 167 (18%) respectively **Fig 2** and **S3 Table.**

At month 12, among the 75 infants stunted at birth, 33/75 (44%) were stunted. The fraction of stunting at months 3, 6 and 12 attributable to stunting at birth was 9.5% (95%CI 5.5 to 13%), 11% (95%CI 6.5 to 15%) and 11% (95%CI 5.0 to 16%) respectively. The fraction of stunting at months 6 and 12 attributable to stunting at month 3 was 51% (95%CI 42 to 59%) and 32% (95%CI 22 to 41%) respectively. The fraction of months 12 stunting attributable to stunting at month 6 was 40% (95%CI 31 to 49%).

### Structural equation models

Confirmatory factor analysis results are presented in **S2 Table**. The child follow-up characteristics latent variable at months 3, 6 and 12 was dominated by attendance of health facility for treatment, a proxy for any episodes of illness and presence of fever. The maternal characteristics latent variable was dominated by maternal age and number of children born, while paternal characteristics were dominated by the fathers’ main economic activity and level of education. The household characteristics latent variable was dominated by assets.

**Fig 3:**
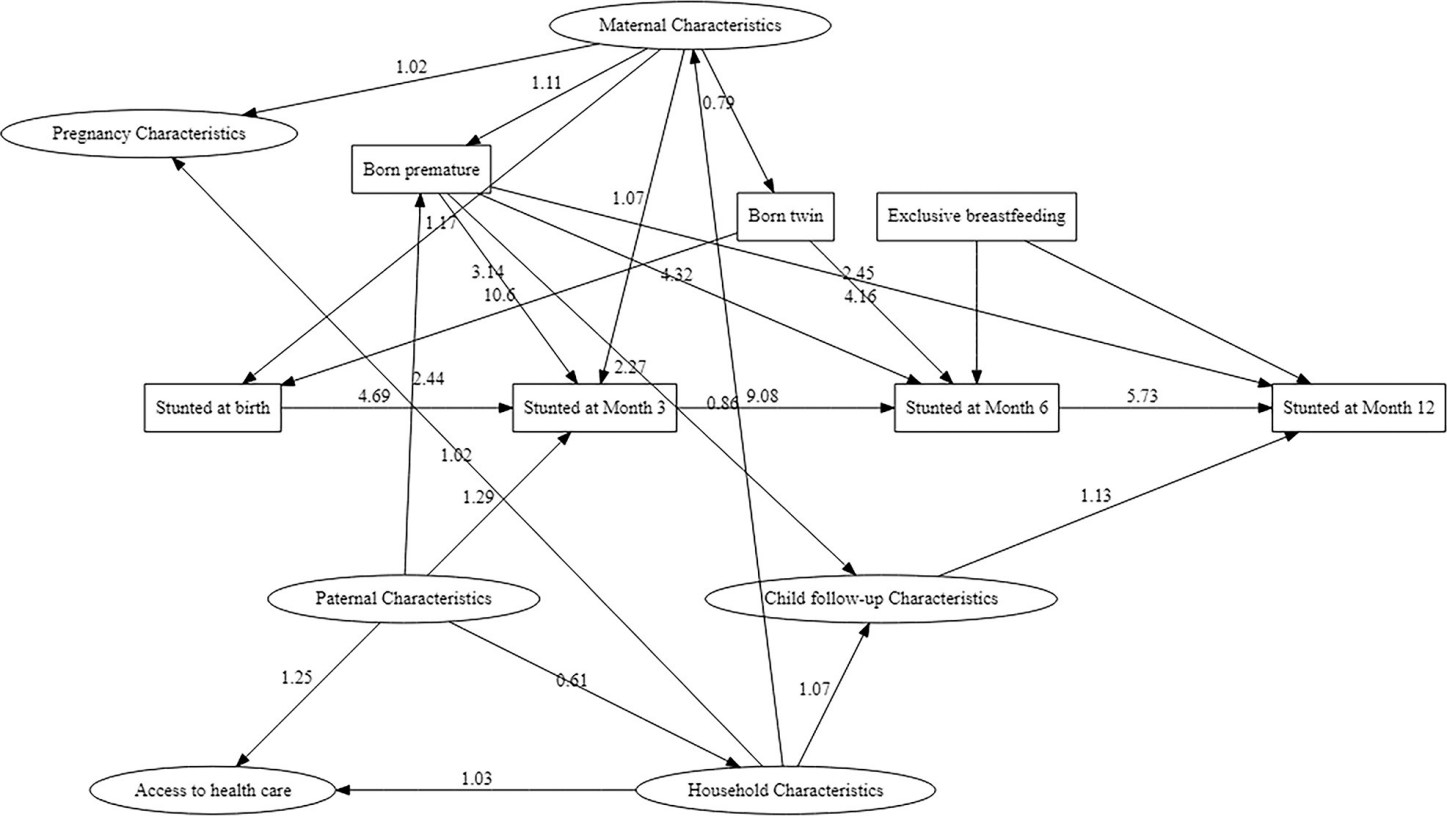
Structural EQUATION Model showing pathways to stunted at birth and months 3, 6 and 12. Only significant pathways (P-value<0.05) are shown. All pathways explored are shown in the conceptual framework in the Supplemental Materials, The arrow thickness is proportional to the effect size. Significant negative effects are indicated by ‒ sign.

In the SEM model, male sex (aOR 1.98 (95%CI 1.19–3.28)), being born a twin (aOR 10.6 (95%CI 4.05–27.9)) and maternal characteristics (aOR 1.17 (95%CI 1.08–1.26)) were directly associated with being stunted at birth. Preterm birth and pregnancy characteristics were not directly associated with being stunted at birth. Indirectly, maternal characteristics influenced being stunted at birth through twin birth. Household characteristics had indirect effects on being stunted at birth through maternal characteristics. Paternal characteristics had an indirect effect mediated through household and maternal characteristics pathway (**Fig 3** and **S5 Table**).

Male sex, being stunted at birth (aOR 4.69 (95%CI 2.63‒8.36)), premature birth and maternal characteristics were directly associated with being stunted at 3 months. Indirectly, being born a twin influenced being stunting at 3 months through being born stunted. Maternal and paternal characteristics continued to have indirect effects through premature birth. Household characteristics continued to have an indirect effect through maternal characteristics. Other pathways are shown in **Fig 3** and **S5 Table.**

Being stunted at birth (aOR 3.98 (95%CI 2.08‒7.58)), stunted at month 3 (aOR 9.08 (95%CI 5.87‒14.0)), being born premature and being born a twin were directly associated with being stunting at month 6. Non-exclusive breastfeeding for six months had direct borderline effect (aOR 1.50 (95%CI 0.93‒2.40)) on being stunting at month 6. Maternal and paternal characteristics continued to have indirect effects through premature birth with maternal effects also via being born a twin and being stunted at month 3. Other pathways are shown in **Fig 3** and **S5 Table.**

Male sex, being stunted at birth, at month 3 and at month 6 (aOR 5.73 (95%CI 3.55‒9.23)), premature birth and child follow-up characteristics (aOR 1.13 (95%CI 1.03‒1.24)) were directly associated with being stunting at month 12. Exclusive breastfeeding had no direct effect on stunting at month 12. Maternal and paternal characteristics continued to have indirect effects through premature birth, with maternal effects also via being stunted at birth, at month 3 and child follow-up characteristics. Household characteristics had indirect effects through child follow-up characteristics. Other pathways are shown in **Fig 3** and **S5 Table.**

## Discussion

In this birth cohort, pathway analysis was applied to determine the direct and indirect pathways to being stunted at birth, 3, 6 and 12 months of age. Not surprisingly, being stunted at birth was directly associated with subsequent stunting. Prematurity was also directly associated with being stunted throughout infancy. Exclusive breastfeeding was directly associated with stunting in early infancy, but not at 12 months. Maternal and household characteristics were indirectly associated with stunting through different pathways. For example at birth, maternal characteristics were associated with stunting through being born a twin pathway while the association with stunting at 3, 6 and 12 months was predominantly through prematurity pathway.

Our findings on the direct association between stunting in infancy with prematurity support the need for prenatal interventions to improve birth outcomes [26, 27] while findings on the indirect association between stunting and maternal characteristics, reveal the roles of both maternal and paternal characteristics during pregnancy and post-delivery period. This pathway association is plausible given sub-optimal breastfeeding has been associated with increased episodes of illness such as diarrhoea and pneumonia in the first year of life [28] and linear growth deficit both among community and hospitalized children [29–31]. Hence our results suggest that exclusively breastfeeding infants for the first 6 months of life will also impact stunting.

Importantly, the family unit and household are important driving factors for stunting, beyond the maternal-infant dyad. Our study identified pathways that are important to differentiate the significance of different drivers of stuntedness at different stages of infancy. The identification of these pathways is an important finding and although the associations may not be new by themselves as others have shown child, maternal, household and paternal characteristics to be important driving factors for stunting [10, 32], our findings uniqueness is in unpacking the complex pathways by which child, maternal, household, and paternal characteristics are associated with being stunted at birth, 3, 6 and at 12 months. This is helpful when one considers a more wholistic approach to stunting prevention. In our study, maternal characteristics is an important direct; at birth and month 3 and indirect; through born twin, born premature or born stunted driver of stunting throughout infancy. This multi-layered relationship can be demonstrated as in the case of an adolescent mother (maternal characteristic-maternal age) presenting a direct risk to having small sized infant at birth (child characteristic-premature) [33] who is likely to be stunted at birth and subsequently. In this way, improving maternal age may depend on changes in household characteristics, for example household income (household characteristic), which may itself be influenced by father’s education level (paternal characteristics), may influence early marriages and improve maternal age [34]. This interdependence between factors within multiple level domains (child/maternal/paternal/household) indicates that interventions targeting factors within one domain may not have the desired impact on stunting in infancy and are likely to be highly context specific. Rather, it may be necessary for interventions to include multiple targets of factors in all domains identified within the stunting pathway and tested for their effectiveness.

Our results further seem to align with the hypotheses that a high socio-economic threshold must be attained before stunting can be eliminated [13]. Collectively, these findings may also help to explain why large WASH based trials either alone or with nutrition interventions have been unsuccessful to improve stunting [12]. Our findings support the current paradigm that multiple interventions need to be introduced simultaneously along an identified pathway.

### Strengths and limitations

Being an older dataset of an untreated birth cohort, the data reflects the natural trajectories of nutritional status from birth without modern interventions which would be impossible to replicate now. However, in the time between 2004 and now, several aspects of health care including access to ANC, maternal nutrition status, education, household assets, coverage of childhood vaccines, malaria control and nutrition programmes have changed which may alter associations.

Within the dataset, gestational age and pregnancy maturation was estimated using the last menstrual period and morphological criteria of foetal maturation. These methods are known to overestimate the duration of gestation classifying more births as post-term than ultrasound scan (4.0% vs 0.7%) [35, 36]. As the study was not originally designed for this analysis, we did not have all desired variables for each domain, for example information on maternal mental health status was not collected. To provide a more comprehensive view of the pathway, future studies, albeit of treated cohorts, should be designed to include all factors described in the WHO conceptual framework for childhood stunting and the further items identified in the present study.

## Conclusions

Direct and indirect pathways identified in this study highlight the complex interlinks between child, maternal, paternal and household characteristics. Overall, our results suggest that interventions to improve birth size such as optimizing nutritional status of women of reproductive age ≥3 months before conception [37, 38] and in the first trimester during pregnancy, socio-economic improvements and exclusive breastfeeding are key targets. Applying a pathways analysis to examine common and divergent pathways of association for wasting, stunting and underweight could help align research and support collaboration across their respective communities of practice that are likely to have much more in common than currently siloed strategies and practice would suggest.

## Supporting information

S1 Table Individual variables used to construct the latent variables. (DOCX)

S2 Table Confirmatory factor analysis of the latent variables. (DOCX)

S3 Table Monthly length-for-age z-score and proportion of children stunting. (DOCX)

S4 Table Comparison of infants and maternal characteristics at time of birth and during follow-up between 940 and 77 assessed and not assessed at month 12. (DOCX)

S5 Table Structural equation modelling analysis of the pathways to stunted at birth. (XLSX)

S1 Fig Structural equation model conceptual framework. (TIFF)

S2 Fig Proportion of events reported during a) ANC visits by the mother and b) one year follow-up of the child.
(TIFF)
