# Supplementary material for: Stunting in the first year of life: Pathway analysis of a birth cohort
Source: PLOS Glob Public Health. 2024 Feb 16;4(2):e0002908. doi: 10.1371/journal.pgph.0002908 (PMC10871522; doi:10.1371/journal.pgph.0002908)
Supplement: S1 Table — (DOCX) [file pgph.0002908.s001.docx]

**S1 Table.** **Individual variables used to construct the latent variables.**

|  | **Stunted at birth** | **Stunting at month 3, 6 & 12** |
| --- | --- | --- |
| **Individual variables included in multivariable model** | | |
| Child sex | BIN | BIN |
| Born twin | BIN | BIN |
| Born premature (gestational age <37weeks) | BIN | BIN |
| Exclusive breastfeeding in first six months | - | BIN |
| **Domain scores** | | |
| **Access to health care** |  |  |
| Place of birth | CAT | CAT |
| ANC attendance | CAT | CAT |
| Distance to market | Log CONT | Log CONT |
| Distance to nearest health facility | Log CONT | Log CONT |
| **Household characteristics** |  |  |
| Presence of toilet in compound | BIN | BIN |
| Water source | BIN | BIN |
| Assets tertiles | CAT | CAT |
| Father source of income | BIN | BIN |
| Household owns a plough | BIN | BIN |
| Type of house wall | BIN | BIN |
| **Maternal characteristics** |  |  |
| Mother age | CAT | CAT |
| Marital status | BIN | BIN |
| Number previous births | CAT | CAT |
| Mother education level | CAT | CAT |
| Mother height | CONT | CONT |
| **Paternal characteristics** |  |  |
| Father age | BIN | BIN |
| Father education level | CAT | CAT |
| Father BMI | CONT | CONT |
| Father main economic activity | BIN | BIN |
| **Pregnancy characteristics** |  |  |
| Albuminuria during pregnancy | BIN | BIN |
| UTI during pregnancy | BIN | BIN |
| Diarrhea during pregnancy | BIN | BIN |
| Fever during pregnancy | BIN | BIN |
| LRTI during pregnancy | BIN | BIN |
| Received Chloroquine during pregnancy | BIN | BIN |
| **Follow-up child characteristics** |  |  |
| Diarrhea during 12 months follow-up | - | BIN |
| Fever during 12 months follow-up | - | BIN |
| Any disease during 12 months follow-up | - | BIN |
| Use of modern health care during 12 months follow-up | - | BIN |
| *Log CONT- natural logarithm of Continuous variable.  †BIN-Binary.  ‡CAT-Categorical. | | |
