# Supplementary material for: Stunting in the first year of life: Pathway analysis of a birth cohort
Source: PLOS Glob Public Health. 2024 Feb 16;4(2):e0002908. doi: 10.1371/journal.pgph.0002908 (PMC10871522; doi:10.1371/journal.pgph.0002908)
Supplement: S2 Table — (DOCX) [file pgph.0002908.s002.docx]

**S2 Table. Confirmatory Factor Analysis of the latent variables.**

|  | **factor loading** | **95% CI** | ***P*** |
| --- | --- | --- | --- |
| **Access to health care *→*** | | | |
| Place of birth | 1.83 | 1.32–2.33 | <0.001 |
| ANC attendance | 0.41 | 0.26–0.57 | <0.001 |
| Distance to market | 0.47 | 0.37–0.57 | <0.001 |
| Distance to nearest health facility | 1 | (constrained) |  |
| **Household characteristics *→*** | | | |
| Presence of toilet in compound | -0.16 | -0.23 to -0.09 | <0.001 |
| Water source | -0.13 | -0.22 to -0.03 | <0.001 |
| Assets tertiles | 1 | (constrained) |  |
| Household owns a plough | 2.14 | -0.25 to 4.53 | 0.08 |
| Type of house wall | -0.17 | -0.25 to -0.08 | <0.001 |
| Father source of income | -0.19 | -0.30 to -0.07 | 0.002 |
| **Maternal characteristics *→*** | | | |
| Mother age | 1 | (constrained) |  |
| Marital status | 0.18 | 0.08 to 0.28 | 0.001 |
| Number previous pregnancies/births | 0.89 | 0.16 to 1.63 | 0.02 |
| Mother education level | 0.02 | -0.03 to 0.06 | 0.45 |
| Mother height | -0.21 | -0.38 to -0.04 | 0.02 |
| **Pregnancy characteristics *→*** | | | |
| Albuminuria during pregnancy | 0.82 | 0.25 to 1.39 | 0.005 |
| UTI during pregnancy | 1 | (constrained) |  |
| Diarrhea during pregnancy | 1.29 | 0.43 to 2.14 | 0.003 |
| Fever during pregnancy | -1.48 | -2.50 to -0.37 | 0.009 |
| LRTI during pregnancy | -1.38 | -2.55 to -0.20 | 0.02 |
| Received Chloroquine during pregnancy | 2.30 | -0.09 to 4.70 | 0.06 |
| **Paternal characteristics *→*** |  |  |  |
| Father age | -0.64 | -1.22 to -0.06 | 0.03 |
| Father education level | 1.58 | -0.98 to 4.14 | 0.23 |
| Father BMI | -0.46 | -0.90 to -0.02 | 0.04 |
| Father main economic activity | 1 | (constrained) |  |
| **Child follow-up characteristics (from birth to month 12) *→*** |  |  |  |
| Diarrhea during 12 months follow-up | 0.60 | 0.43 to 0.77 | <0.001 |
| Fever during 12 months follow-up | 1 | (constrained) |  |
| Any disease during 12 months follow-up | 0.40 | 0.27 to 0.52 | <0.001 |
| Use of modern health care during 12 months follow-up | 0.81 | 0.56 to 1.06 | <0.001 |
| Notes: Confirmatory Factor Analysis results, where each domain was estimated separately as a latent variable that gives rise to the observed underlying measures of the respective domain. The strongest factor loading of a domain is the best measure of that domain. The constrained factor loading (set to 1) in each domain was chosen using exploratory factor analysis. | | | |
