# Supplementary material for: Stunting in the first year of life: Pathway analysis of a birth cohort
Source: PLOS Glob Public Health. 2024 Feb 16;4(2):e0002908. doi: 10.1371/journal.pgph.0002908 (PMC10871522; doi:10.1371/journal.pgph.0002908)
Supplement: S3 Table — (DOCX) [file pgph.0002908.s003.docx]

**S3 Table. Monthly Length-for-age z-score and proportion of children stunting.**

|  | **Birth** | **Month 1** | **Month 2** | **Month 3** | **Month 4** | **Month 5** | **Month 6** | **Month 7** | **Month 8** | **Month 9** | **Month 10** | **Month 11** | **Month 12** |
| --- | --- | --- | --- | --- | --- | --- | --- | --- | --- | --- | --- | --- | --- |
| Monthly LAZ and stunting levels | | | | | | | | | | | | | |
| N | 1017 | 883 | 880 | 937 | 898 | 883 | 935 | 889 | 898 | 928 | 894 | 884 | 940 |
| Mean LAZ ±sd | -0.18 ±1.2 | -0.29±1.6 | -0.62 ±1.7 | -0.72 ±1.8 | -0.71 ±1.7 | -0.67 ±1.6 | -0.77 ±1.6 | -0.81 ±1.5 | -0.88 ±1.4 | -0.95 ±1.3 | -1.02 ±1.3 | -1.15 ±1.2 | -1.18 ±1.2 |
| Stunted (LAZ<-2) | 75 (7.4) | 101 (11) | 172 (20) | 214 (23) | 164 (18) | 169 (19) | 187 (20) | 164 (18) | 195 (22) | 181 (20) | 172 (19) | 171 (19) | 167 (18) |
| Other anthropometric measurements | | | | | | | | | | | | | |
| Mean Length ±sd | 49.0 ±2.4 | 53.4 ±3.2 | 56.5 ±3.4 | 59.1 ±3.7 | 61.5 ±3.7 | 63.5 ±3.6 | 65.1 ±3.5 | 66.4 ±3.4 | 67.7 ±3.3 | 68.9 ±3.3 | 70.0 ±3.0 | 70.9 ±2.9 | 72.0 ±2.8 |
| Mean Weight ±sd | 2.84 ±0.4 | 4.00 ±0.7 | 4.91 ±0.8 | 5.60 ±0.9 | 6.08 ±0.9 | 6.40 ±0.9 | 6.66 ±0.9 | 6.88 ±1.0 | 7.06 ±1.0 | 7.25 ±1.0 | 7.42 ±1.0 | 7.60 ±1.0 | 7.84 ±1.0 |
| Mean MUAC ±sd | 10.3 ±1.0 | 11.7 ±1.3 | 12.6 ±1.3 | 13.3 ±1.3 | 13.6 ±1.4 | 13.7 ±1.4 | 13.7 ±1.4 | 13.7 ±1.5 | 13.7 ±1.5 | 13.7 ±1.4 | 13.7 ±1.4 | 13.7 ±1.5 | 13.9 ±1.5 |

LAZ-Length-for-age z-score, sd-Standard deviation.
