# Supplementary material for: Stunting in the first year of life: Pathway analysis of a birth cohort
Source: PLOS Glob Public Health. 2024 Feb 16;4(2):e0002908. doi: 10.1371/journal.pgph.0002908 (PMC10871522; doi:10.1371/journal.pgph.0002908)
Supplement: S4 Table — (DOCX) [file pgph.0002908.s004.docx]

S4 Table. Comparison of infants and maternal characteristics at time of birth and during follow-up between 940 and 77 assessed and not assessed at month 12.

|  | **Infants assessed in this study (N=940)** | **Excluded infants (N=77)** | **P-value** |
| --- | --- | --- | --- |
| **Demographics** |  |  |  |
| Sex |  |  |  |
| Male | 482 (51) | 40 (52) | 0.91 |
| Gestation age in weeks; median [IQR] | 38.9 (38.2–40.2) | 38.9 (37.5–40.2) | 0.42 |
| Born premature* | 43 (4.6) | 5 (6.5) | 0.45 |
| Born twins | 25 (2.7) | 3 (3.9) | 0.52 |
| Born low birth weight^#^ | 179 (19) | 10 (13) | 0.19 |
| Place of birth |  |  |  |
| Health facility | 419 (45) | 35 (45) |  |
| Home with CBA | 371 (39) | 28 (36) | 0.82 |
| Home with no CBA | 150 (16) | 14 (18) |  |
| Recruitment health centre |  |  |  |
| Basma | 350 (37) | 37 (48) |  |
| CMA | 282 (30) | 19 (25) | 0.28 |
| Dablo | 237 (25) | 15 (19) |  |
| Foube | 71 (7.6) | 6 (7.8) |  |
| **Birth anthropometry (**mean ±sd) |  |  |  |
| Length (cm), mean ±sd | 49.0 ± 2.4 | 49.1 ± 2.4 | 0.60 |
| Weight (kg); mean ±sd | 2.8 ± 0.4 | 2.8 ± 0.4 | 0.69 |
| MUAC in cm; mean ±sd | 10.3 ± 1.1 | 10.1 ± 0.9 | 0.24 |
| LAZ; mean ±sd | -0.19 ± 1.2 | -0.07 ± 1.3 | 0.42 |
| WLZ; mean ±sd | -1.34 ± 1.5 | -1.45 ± 1.8 | 0.55 |
| WAZ; mean ±sd | -0.94 ± 0.9 | -0.94 ± 1.0 | 0.99 |
| Stunted; N (%, 95%CI) | 68 (7.2) | 7 (9.1) | 0.55 |
| Underweight; N (%, 95%CI) | 115 (12) | 7 (9.1) | 0.41 |
| Wasted; N (%, 95%CI) | 280 (30) | 23 (30) | 0.98 |
| **Child follow-up characteristics** |  |  |  |
| Exclusive breastfeeding |  |  |  |
| ≥3 months | 710 (76) | 32 (42) |  |
| Up to 3 months | 226 (24) | 20 (26) | 0.18 |
| None from birth | 26 (2.8) | 3 (3.9) |  |
| Follow-up illness (No. of children with at least one episode) |  |  |  |
| Fever | 693(73) | 48 (62) | 0.24 |
| Diarrhoea | 453 (48) | 30 (39) | 0.09 |
| Cough | 479 (51) | 42 (55) | 0.84 |
| **Maternal characteristics** |  |  |  |
| Mother age; median (IQR) years | 25 (20−30) | 22 (18−28) | 0.08 |
| Illiterate | 718 (76) | 61 (79) | 0.82 |
| Number of ANC visits |  |  |  |
| None | 49 (5.2) | 3 (3.9) |  |
| 1 to 3 | 873 (93) | 74 (96) | 0.41 |
| ≥ 4 | 18 (1.9) | 0 |  |
| Mother height at delivery; median (IQR) cm | 163 (159 to 168) | 164 (159 to 168) | 0.86 |
| **Paternal** **characteristics** |  |  |  |
| Father age; median (IQR) years | 39 (31 to 50) | 39 (33 to 45) | 0.62 |
| Father illiterate | 476 (52) | 38 (58) | 0.76 |
| Father Body Mass Index (BMI) |  |  |  |
| <18.5 | 95 (10) | 3 (3.9) |  |
| 18.5 to 25 | 758 (81) | 71 (92) | 0.14 |
| ≥25.0 | 87 (9.3) | 3 (3.9) |  |
| **Household characteristics** |  |  |  |
| Wealth quantiles |  |  |  |
| Quintile 1 (most assets) | 193 (21) | 14 (18) |  |
| Quintile 2 | 192 (20) | 15 (19) |  |
| Quintile 3 | 188 (20) | 13 (17) | 0.12 |
| Quintile 4 | 201 (21) | 12 (16) |  |
| Quintile 5 (Least assets) | 166 (18) | 23 (30) |  |
| Distance to nearest health facility (km) |  |  |  |
| < 5Km | 348 (37) | 26 (34) | 0.57 |
| ≥ 5Km | 592 (63) | 51 (66) |  |
| *gestational age <37 weeks, #birth weight <2.5kg, CBA: community based assistant, MUAC: mid-upper arm circumference, LAZ: length-for-age z-score, WLZ: weight-for-length z-score, WAZ: weight-for-age z-score, sd: standard deviation, results are N (%) unless specified, asserts used to calculate wealth quantiles are: bicycle, motorcycle, car, cart, plough, donkey, goat, sheep, pig, cow, radio, TV, type of the house (mud or bricks). | | | |
