# Supplementary figures and images for: Stunting in the first year of life: Pathway analysis of a birth cohort
Source: PLOS Glob Public Health. 2024 Feb 16;4(2):e0002908. doi: 10.1371/journal.pgph.0002908 (PMC10871522; doi:10.1371/journal.pgph.0002908)

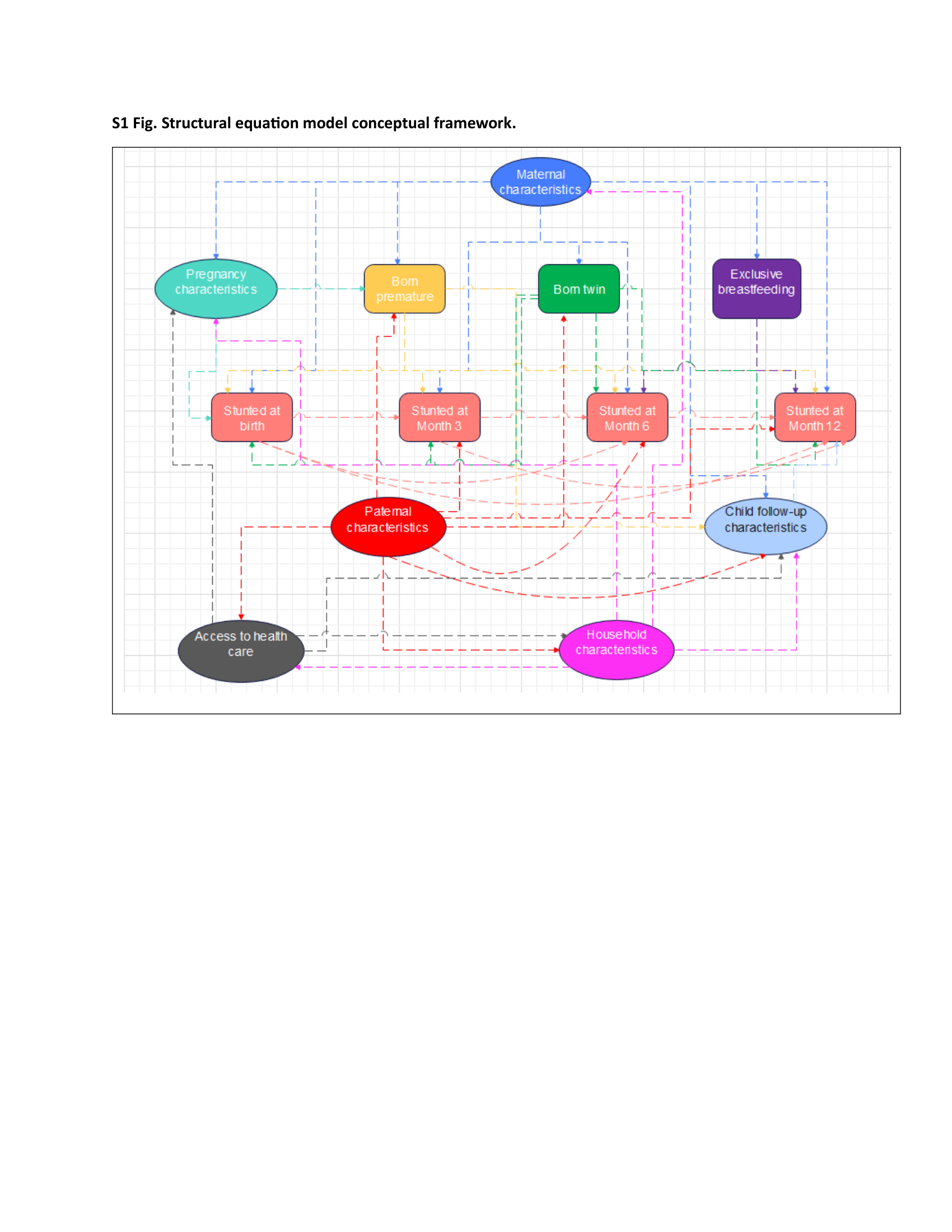

Supplement: S1 Fig — (TIFF) [file pgph.0002908.s006.tiff]

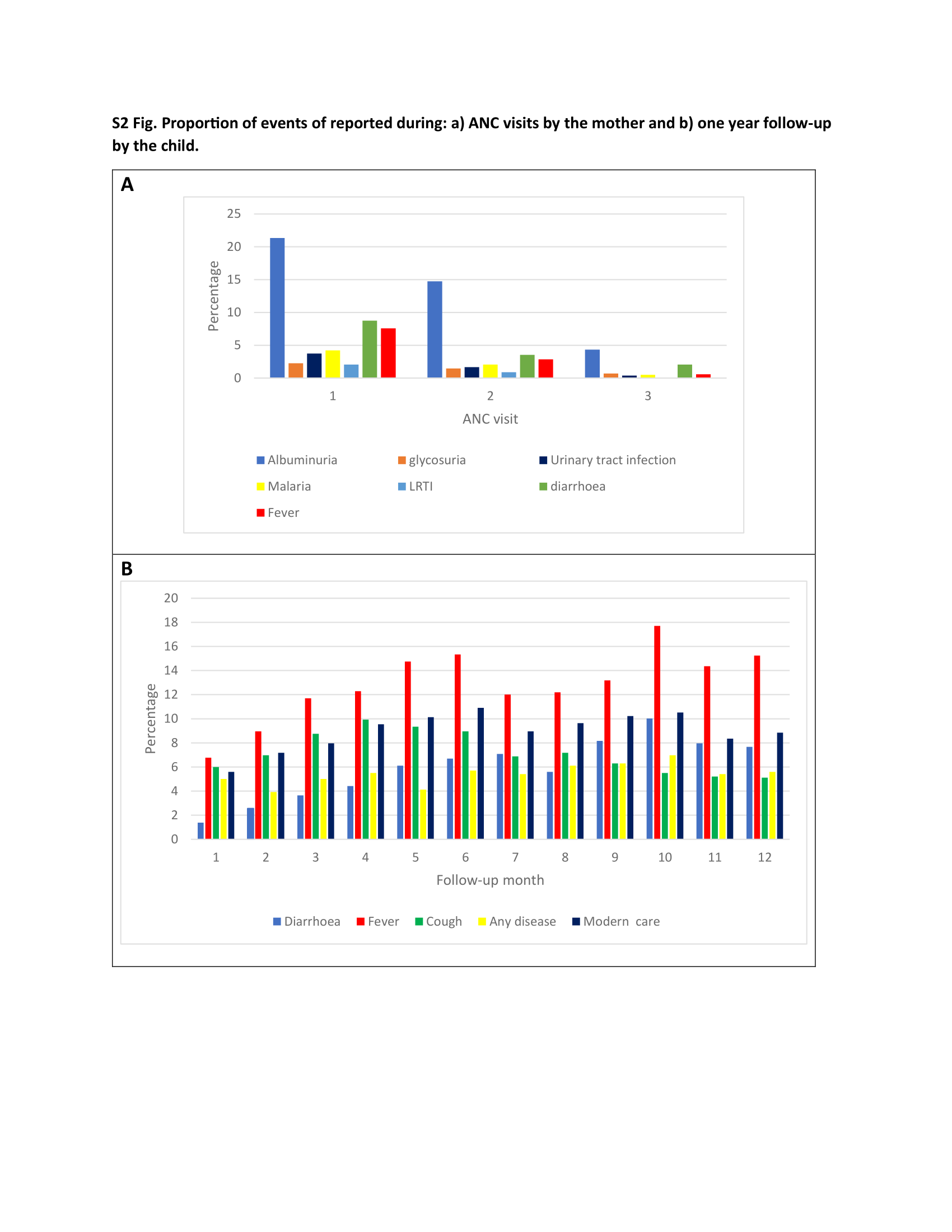

Supplement: S2 Fig — Proportion of events reported during a) ANC visits by the mother and b) one year follow-up of the child. (TIFF) [file pgph.0002908.s007.tiff]
